# Neural Net Gains Estimation Based on an Equivalent Model

**DOI:** 10.1155/2016/1690924

**Published:** 2016-06-05

**Authors:** Karen Alicia Aguilar Cruz, José de Jesús Medel Juárez, José Luis Fernández Muñoz, Midory Esmeralda Vigueras Velázquez

**Affiliations:** ^1^Centro de Investigación en Computación, Instituto Politécnico Nacional (CIC-IPN), Avenida Juan de Dios Bátiz, Esq. Miguel Othón de Mendizábal, Col. Nueva Industrial Vallejo, Delegación Gustavo A. Madero, 07738 Ciudad de México, DF, Mexico; ^2^Centro de Investigación en Ciencia Aplicada y Tecnología Avanzada U. Legaria, Instituto Politécnico Nacional (CICATA-Legaria-IPN), Calzada Legaria 649, Col. Irrigación, Delegación Miguel Hidalgo, 11500 Ciudad de México, DF, Mexico

## Abstract

A model of an Equivalent Artificial Neural Net (EANN) describes the gains set, viewed as parameters in a layer, and this consideration is a reproducible process, applicable to a neuron in a neural net (NN). The EANN helps to estimate the NN gains or parameters, so we propose two methods to determine them. The first considers a fuzzy inference combined with the traditional Kalman filter, obtaining the equivalent model and estimating in a fuzzy sense the gains matrix *A* and the proper gain *K* into the traditional filter identification. The second develops a direct estimation in state space, describing an EANN using the expected value and the recursive description of the gains estimation. Finally, a comparison of both descriptions is performed; highlighting the analytical method describes the neural net coefficients in a direct form, whereas the other technique requires selecting into the Knowledge Base (KB) the factors based on the functional error and the reference signal built with the past information of the system.

## 1. Introduction

There are different techniques for modelling a system to identify the characteristics that make it an excellent approximation. When considering the physical behaviour of the real process, these models usually lead to complex nonlinear systems, which are difficult to analyse and are simplified through relations between their input and output signals, obtaining a Black-Box (BB) description [1].

The system evolution viewed as a BB has no access to its internal properties but only to the input and output responses, without paying attention to the internal parameters that have a dynamical evolution. However, the human experiences, in many cases, give the original answer or selection (a fair process) through the intuition (if then (fuzzy logic) inferences) selecting the new parameters. These experiences provide better tolerable approximations in combination with other theories, for example, Lyapunov, Sliding Modes, or Intelligent Systems.

Artificial Neural Nets (ANN) viewed as mathematical models, applied for complex systems [2, 3], and inspired by biological neurons operations, generate a fast answer. Nevertheless, the computer devices, compared with the human brain, have different connotations because the methods considered are faster due to robust programming instead of chemical reactions in natural conditions. In addition, the human brain has the ability of self-programming and adaptability without requiring a new programming code, adjusting its required energy levels based on natural instincts using the intuition as a great tool.

**Figure 1:**
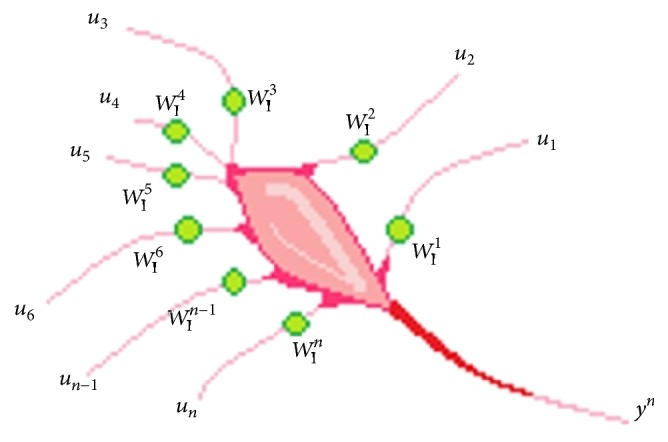
Basic neuron model.

An artificial neural model (Figure 1) based on a biological neuron principle implemented in a computational model is helpful in prediction and classification of problems, pattern recognition, signal processing, estimation, and control [4]. In the case when more than a neuron is connected, we obtain a MISO (Multiple Inputs and a Single Output) neural net, and similar to human adaptability, the model considered adjusts its functional parameters using learning processes for different outputs depending on the stimuli.

When analysing a system, its characteristics help us determine the best method according to our requirements, improving the convergence rate. In this paper, we compare a hybrid model and an analytical model. The first implies a fuzzy estimation combined with the Kalman filter description, and the second is optimal with systematic evaluation, considering the expected value obtained from the previous information. Both are based on an Equivalent Artificial Neural Net (EANN).

## 2. Equivalent Neural Net

In the biological sense, for neural-specific tasks, the neuron cell inputs require accomplishing adequate properties for generating a fire action in the neuron soma to obtain an accurate output in the neuron axon; in other cases, the biological system operates with the minimal energy required which allows not losing connection with other neurons.

**Figure 2:**
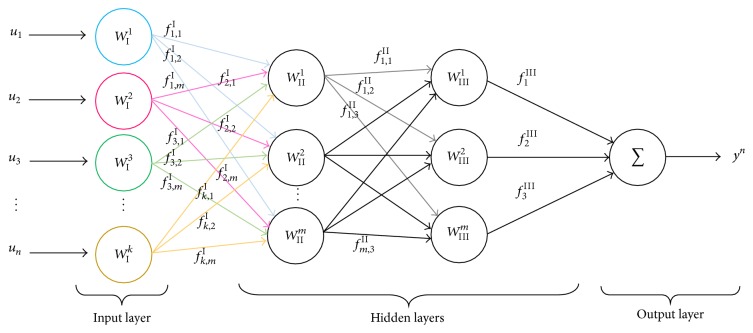
Artificial Neural Net description.

In an ANN, we identify three principal sections: input, hidden, and output layers. The input layer is the interaction between the input signals and the first block of weights; its result becomes the input to the following coating. At the very first stage, the designer selects the weights intuitively and adjusts the following while looking for the desired response [5]. The hidden layers have a set of inputs and outputs in different stages related through the weights. The output layer represents the convolution or binary sum of the last block of weights and its respective data. In an illustrative way, Figure 2 depicts an example of a typical ANN, with two hidden layers, input and output.

The EANN is a simplified representation of an ANN whose task is to obtain the parameters vector which allows the system to reach the desired reference signal without paying initial attention to the internal layers, focusing on the estimation procedure. It considers the total input-output signals relation trying to reduce unnecessary delays and always keeping the weight interconnection form, achieving the desired response.

**Figure 3:**
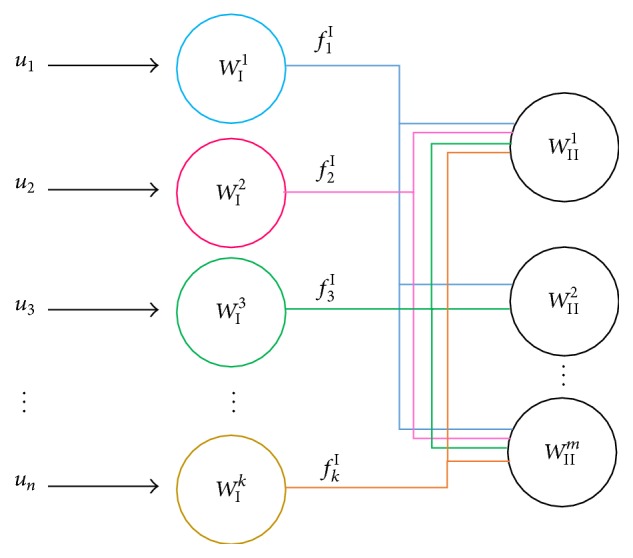
Simple description of an Artificial Neural Net through activation functions.

As shown in Figure 2, input data is denoted by a set described as $\left\{u_{i}:i=\overset{-}{1,n},\;\;n\in Z_{+},\;\;u\in R\right\}$, and the output data is denoted by {*y* ^*n*^ ∈ *R*}, where *n* represents the number of input elements. In addition, the weights or parameters are considered as ${\left\{W_{l}^{k}:k=\overset{-}{1,n},\;\;n,l\in Z_{+}\right\}}_{l=\overset{-}{1,L}}$, where *k* indicates the specific parameter number in a layer *l*. The admitted layers are within a set of functions ${\left\{{\left\{f_{i,j}^{l},\;\;j=\overset{-}{1,m},\;\;m\in Z_{+}\right\}}_{i=\overset{-}{1,n}}\right\}}_{l=\overset{-}{1,L}}$, interconnecting directionally from an original parameter *i* into layer *l* to a target parameter *j* into layer (*l* + 1). Each weight from the input and output layers requires an activation function and all the hidden layers have proper activation functions connected to other weights to achieve different and specific requirements for each output stage. This description corresponds to Figure 3, where the traditional ANN connections have now simple flow diagram lines containing activation functions described as $\left\{{\left\{f_{i}^{l},\;\;i=\overset{-}{1,n};\;\;n,l\in Z_{+}\right\}}_{l=\overset{-}{1,L}}\right\}$, where *i* represents the function number and *l* represents the layer this functions leaves.

Figure 3 presents the activation functions from the first hidden layer (*l* = I) operating with an accumulative energy *W*_I_^*k*=*i*^ convolved with an input *u*_*i*_ in agreement with the following equation:

(1) $$\begin{array}{c} f_{i}^{I}=\left\{\begin{array}{cc} W_{I}^{i}\circ u_{i} & \text{if }\underline{\varpi_{i}}\leq \left|u_{i}\right|\leq \bar{\varpi_{i}}\\ 0 & in\;\;other\;\;cases. \end{array}\right. \end{array}$$

The set of pairs $\left\{\left(\underline{\varpi_{i}},\bar{\varpi_{i}}\right):i=\overset{-}{1,n},\;\;n\in Z_{+}\right\}$ represents activation limit functions for *f*_*i*_^I^. These limits denote the minimum and maximum required energy to excite a neuron for a specific weight *W*_I_^*i*^, known as fire limits. In the following hidden layer (II), *f*_*i*_^II^ requires that the set of inputs accomplishes the same requirements, considered for previous results; that is,

(2) $$\begin{array}{c} f_{i}^{II}=\left\{\begin{array}{cc} W_{II}^{i}\circ \left(f_{1}^{I}*f_{2}^{I}*\cdots *f_{n}^{I}\right) & \text{if }\underline{\varpi_{i}^{II}}\leq \left|\left(f_{1}^{I}*f_{2}^{I}*\cdots *f_{n}^{I}\right)\right|\leq \bar{\varpi_{i}^{II}}\\ 0 & in\;\;other\;\;cases. \end{array}\right. \end{array}$$

In (2), the binary operator “*∗*” represents the composition of the involved terms without indicating a particular operation.

**Figure 4:**
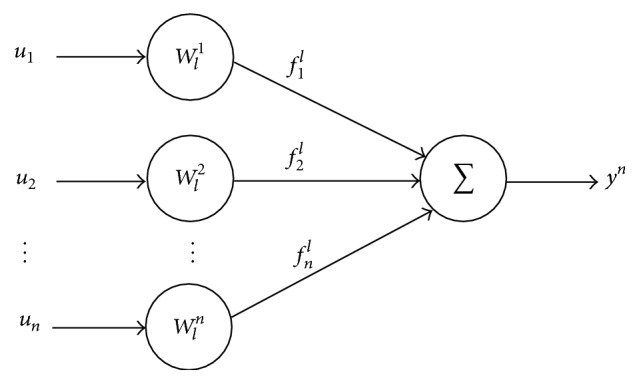
Equivalent Neural Net representation.

The equivalent weights sequence allows each input to include the structure of the previous parameters in the final description. Each layer takes part in the following activation function due to the interaction between the new weights and the previous composed output signal.  Figure 4 shows the EANN model in the simplified form.

According to [6], each neuron output has a function whose parameters are the inputs and weights for the following layer. Equation (3) expresses the influence of the previously mentioned parameters, weights, and inputs to the following layer in a recursive form, where, instead of *y* ^*n*^, *S*_*l*_^*n*^ describes the operation ∑_*i*=1_^*n*^ *f*_*i*_^*l*^ *W*_*l*_^*i*^ *u*_*i*_ as the core neuron function:

(3) $$\begin{array}{c} S_{l}^{n}=f_{n}^{l}W_{l}^{n}u_{n}+{MS}_{l}^{n-1}, \end{array}$$

where *M* is a proportional constant adjusting the previous layer; therefore, the model converges to the neural net development. At the final coat, we have the convolution *y* ^*n*^ = ((*F*∘*W*)∘*U*)_*l*_^*n*^, which represents the neural net response. For computational applications, this reaction has the effect of an activation function, usually the sigmoid function.

A sophisticated ANN considers the integration of more than one EANN since its description allows using the recursive characteristics. In addition to this, the implementation of EANNs gives the possibility to restrict the number of necessary iterations to reach a reference, which is the remarkable feature in systems where time delays are considerable.

## 3. Equivalent Neural Net Using Arma Description

An ARMA (1,1) (Autoregressive Moving Average) model is a tool used for obtaining the parameters matrix from a reference system viewed as a MISO BB; its primary structure is specified by (4) and (5), with *n* being the time evolution:

(4) Sln+1=ASln+Blnun,

(5) yn=CSln,

where *S*_*l*_^*n*^ ∈ *R*_[0,1_, {*u*_*n*_}⊆*N*(*μ*_*u*_, *σ*_*u*_^2^ < *∞*), and *y* ^*n*^ ∈ *R*_[0,1_.

This model has observable (*y* ^*n*^) and internal (*S*_*l*_^*n*^) states, an input signal (*u*_*n*_), gains (*B*, *C*), and internal gain (*A*). The measurable state (6) in explicit form is a function of its immediate past, internal gain, and the inputs ${\left\{u_{i}\right\}}_{i=\overset{-}{1,n}}$. Consider

(6) $$\begin{array}{c} y^{n}=f\left(\;y^{n-1},A,{\left\{\;u_{i}\;\right\}}_{i=\overset{-}{1,n}}\;\right). \end{array}$$

In [7] the internal state using the traditional Kalman filter (KF) is described even though the internal gain *A* and the gain *K* ^*n*^ are still unknown. The complexity of the filter increases because after the identification the internal gain depends on the error, which has an application in (4) for obtaining the observable signal approximation in (5), represented in the discrete form in the following equation:

(7) $$\begin{array}{c} S_{l}^{n}=S_{l}^{n-1}{+B}_{l}^{n-1}u_{n-1}. \end{array}$$

By applying (7) in (4), including the still unknown internal state, we obtain (8)

(8) $$\begin{array}{c} y^{n}=C{AS}_{l}^{n-1}+{CB}_{l}^{n-1}u_{n-1}. \end{array}$$

The internal state from (6) allows in (9) obtaining the internal lagged state as a measurable state function and output perturbations. Consider

(9) $$\begin{array}{c} S_{l}^{n-1}=C^{+}y^{n-1}. \end{array}$$

Considering (9) in (8) we determine the output in the following equation:

(10) $$\begin{array}{c} y^{n}=Ay^{n-1}+{CB}_{l}^{n-1}u_{n-1}. \end{array}$$

Equation (11) represents a recursive form of (10) describing the reference system with an innovation process:

(11) yn=Ayn−1+δ^w^n,

(12) δ^w^n=CSln−1un−1.

In agreement with [8], the gain (*K* ^*n*−1^) with (12) corresponds to $K^{n-1}\hat{\delta }{\hat{w}}_{n}\approx {\hat{y}}^{n}-{\hat{A}}_{n}y^{n-1}$. The hybrid filter (13) considers the fuzzy parameter estimation, the gain description, and the lagged signal:

(13) $$\begin{array}{c} {\hat{y}}^{n}={\hat{A}}_{n}y^{n-1}+K^{n-1}\hat{\delta }{\hat{w}}_{n}. \end{array}$$

With the innovation process and the reference system, bounded by the same general Membership Function (MF) [8, 9], it is possible to estimate the explicit matrix parameters and the gain using the inference mechanisms considering the functional results and the noise properties, respectively.

## 4. Fuzzy Gains and Estimation Properties

In the fuzzy sense, [10] presented the parameters obtained by a controller, considering a fuzzy function vector for nonlinear systems. The MIMO system found firstly the linear representation formed by a collection of MISO systems with the same inputs, reducing and simplifying its analysis.

On [11], the hybrid combination required that the identification filter adjust the parameters automatically using fuzzy logic. This adjustment needs the selection of the best values with respect to the inference, minimizing the error convergence by using heuristic techniques or the Least Square Method (LSM).

The first step in the fuzzy estimation determines the reasoning levels in accordance to the proposed MF, identified through the reference signal statistical properties. There could be triangular, sinusoidal, and impulsive or Gaussian functions, among others, to define the ranges contained in the reference signal classification.

A set of fuzzy rules (if then) forms a Fuzzy Rule Base (FRB) to interpret what requirements and process conditions are needed. Previously, it is necessary to select and introduce the best values to the Knowledge Base (KB) according to the MF, actualizing the parameters according to the reference model limited by the filter error criteria.

Using the fuzzy logic connectors into the fuzzy stage, considering the desired signal (*y* ^*n*^) and the region level with respect to (*J*_*n*_^*n*^), reducing the inference operational levels and indicators in to the MF, and selecting from the KB parameters ${\hat{A}}_{n}$ values actualise the hybrid filtering process. Each fuzzy filtering rule finds specific matrix parameters in each evolution [9, 12].

In the same sense, the hybrid filter considers the basic principles of a conventional Kalman digital filter using the Mean Least Square Criterion (MLSC) described as *J*_*n*_^*n*^ = 〈*e* ^*n*^, *e*_*n*_^*T*^〉^(1/2)^ and, in agreement with [5], in its recursive form:

(14) $$\begin{array}{c} J_{n}^{n}={\left[\;e^{n}e_{n}^{T}+J_{n-1}^{n-1}\;\right]}^{1/2}\in R_{\left[0,1\right)}. \end{array}$$

According to [9], (14) presents the adequate element describing the optimal matrix parameters.

## 5. Optimum Coefficient

For an ANN, to determine an optimal vector coefficient is necessary to consider minimizing the error and as the primary objective that the convergence tends to zero. One inconvenience is how long this optimal convergence will take to occur. A control for a recurrent NN, described in [13], was an optimum by adding an extra coefficient to compensate for the error within a small bound in an unknown necessary learning time.

Considering the fact that the last stage of a hybrid filter corresponds to the equivalent neural net from Figure 3, it is possible to determine the optimum parameters for the neural weights obtaining the best output approximation to the reference signal by an analytical process.

Based on BB concepts, the input signals ${\left\{u_{i_{n}}\right\}}_{i=\overset{-}{1,N}}$ represented by the matrix *x* ^*n*^_[*N* × 1]_ and the output of the system *y* ^*n*^ are the known parameters. In this sense, we need a synthesis process to calculate the matrix values *A*_[1 × *N*]_ representing the weights in the neural layer.

Having *y* ^*n*^ = *Ax* ^*n*^ as an ARMA model and the process considering stochastic properties, we use the mathematical expectation in the probabilistic sense obtaining information about the process. So ${\hat{A}}_{n}≔E\left\{y^{n}x_{n}^{T}\right\}{\left[E\left\{x^{n}x_{n}^{T}\right\}\right]}^{+}$, where the symbols *T* and + represent the transpose and pseudoinverse operators, respectively.

If *y* ^*n*^ is the reference signal which helps us get the parameters, then we apply these values to find the output *y* ^iden,*n*^, and their comparison gives the identification error *e* ^*n*^≔*y* ^*n*^ − *y* ^iden,*n*^ and its functional error *J*_*n*_^*n*^≔〈*e* ^*n*^, *e*_*n*_^*T*^〉 tending to zero due to the values being considered optimums.

To demonstrate this, from Figure 4, the output is observed as

(15) $$\begin{array}{c} y^{n}=w_{I}^{1}u_{1_{n}}+w_{I}^{2}u_{2_{n}}+\cdots +w_{I}^{N}u_{N_{n}}. \end{array}$$

In addition, seeing *y* ^*n*^ as the reference or target signal defined as

(16) A≔wI1wI2⋯wIN1×N;xnT≔u1nu2n⋯uNn1×N,

we have the following form:

(17) $$\begin{array}{c} y^{n}=Ax^{n}. \end{array}$$

Considering *x*_*n*_^*T*^ is a stochastic input formed in distribution sense by ${\left\{{\left(u_{i_{n}}\right)}^{T}\subseteq N\left(\mu ,\sigma^{2}<\infty \right)\right\}}_{i=\overset{-}{1,N}}$, the parameters are represented by *A* and the output signal is represented by *y* ^*n*^; the BB system scheme allows estimating the parameters set through its time evolution in a probabilistic sense. Consider

(18) $$\begin{array}{c} E\left\{\;{y^{n}}_{\left[1\right]}x_{n_{\left[1\times N\right]}}^{T}\;\right\}=E\left\{\;A_{\left[1\times N\right]}{x^{n}}_{\left[N\times 1\right]}x_{n_{\left[1\times N\right]}}^{T}\;\right\}. \end{array}$$

Due to the weights being constants for an instant of execution time *n* and considering the mathematical expectation properties, it is possible to obtain the matrix estimation known as ${\hat{A}}_{n}$, indicating that this new array value is the matrix estimation. Consider

(19) $$\begin{array}{c} {\hat{A}}_{n_{\left[1\times N\right]}}≅E\left\{\;{y^{n}}_{\left[1\right]}x_{n_{\left[1\times N\right]}}^{T}\;\right\}{\left(\;E\left\{\;{x^{n}}_{\left[N\times 1\right]}x_{n_{\left[1\times N\right]}}^{T}\;\right\}\;\right)}^{+}. \end{array}$$

For a discrete system (19) with infinite enumerable elements, the mathematical expectation has the following form:

(20) A^n1×N1n∑i=1nyi1xi1×NT1×N·1n∑i=1nxiN×1xi1×NTN×N+.

By replacing ${\hat{A}}_{n_{\left[1\times N\right]}}$ in (17), we obtain a new output state of *y* ^*n*^ which we call identification symbolically described as *y* ^iden,*n*^; it represents the output including the effects of the estimated weights values.

The difference between the identified signal and the reference signal gives the following identified error:

(21) $$\begin{array}{c} e^{n}=y^{n}-y^{\text{iden},n}. \end{array}$$

In order to express (20) recursively, the first and second terms are replaced with *P* ^*n*^ and *Q*_*n*_, respectively, defined as follows:

(22) Pn=1n∑i=1nyixiT,

(23) Qn=1n∑i=1nxixiT.

Considering (22) and (23) in (20), (24) and its delayed form (25) for stable conditions are obtained:

(24) A^n=PnQn+,

(25) A^n−1=Pn−1Qn−1+.

Developing (22) in recursive manner has the following equation:

(26) $$\begin{array}{c} P^{n}=\left(\;\frac{1}{n}\;\right)\left(\;y^{n}x_{n}^{T}+\overset{n-1}{\underset{i=1}{\sum }}y^{i}x_{i}^{T}\;\right). \end{array}$$

Considering stationary conditions for (22) delayed has

(27) $$\begin{array}{c} P^{n-1}=\left(\;\frac{1}{n-1}\;\right)\overset{n-1}{\underset{i=1}{\sum }}y^{i}x_{i}^{T}. \end{array}$$

**Figure 5:**
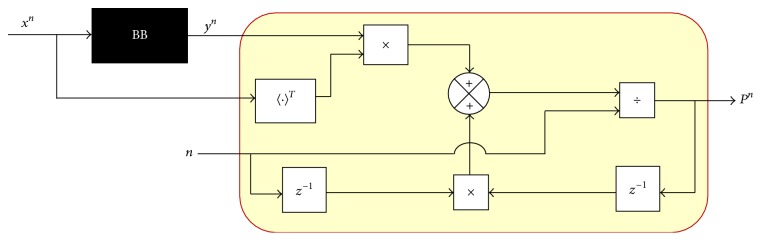
Obtainment of *P* ^*n*^, recursive.

Rewriting (26) in terms of (27), we have (28) and its block diagram representation shown in Figure 5:

(28) $$\begin{array}{c} P^{n}=\left(\;\frac{1}{n}\;\right)\left(\;y^{n}x_{n}^{T}+\left(\;n-1\;\right)P^{n-1}\;\right). \end{array}$$

Expanding (28) and ordering with respect to *P* ^*n*−1^, we have the following equation:

(29) $$\begin{array}{c} P^{n}=\left(\;\frac{n-1}{n}\;\right)P^{n-1}+\left(\;\frac{1}{n}\;\right)y^{n}x_{n}^{T}. \end{array}$$

Now, applying (29) in (24), we have the following estimation:

(30) $$\begin{array}{c} {\hat{A}}_{n}=\left(\;\frac{n-1}{n}\;\right)P^{n-1}Q_{n}^{+}+\left(\;\frac{1}{n}\;\right)y^{n}x_{n}^{T}Q_{n}^{+}. \end{array}$$

Remembering that (25) in stationary conditions is the estimation delayed and applying it in (30) yields the following:

(31) $$\begin{array}{c} {\hat{A}}_{n}=\left(\;\frac{n-1}{n}\;\right){\hat{A}}_{n-1}\left(\;Q_{n-1}Q_{n}^{+}\;\right)+\left(\;\frac{1}{n}\;\right)y^{n}x_{n}^{T}Q_{n}^{+}. \end{array}$$

**Figure 6:**
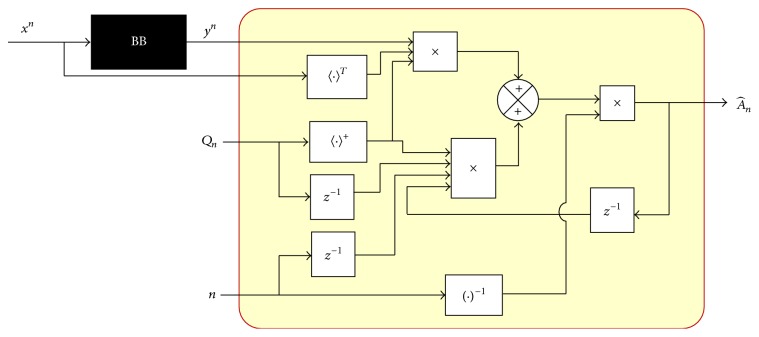
Parameter ${\hat{A}}_{n}$ block diagram.

Using (31) in (24), we obtain the parameter vector in recursive form (32). The block diagram representing ${\hat{A}}_{n}$ parameter using (31) is in Figure 6. Consider

(32) $$\begin{array}{c} {\hat{A}}_{n}={\hat{A}}_{n-1}\alpha^{n-1}+\beta_{n-1}, \end{array}$$

where *α* ^*n*−1^ = ((*n* − 1)/*n*)*Q*_*n*−1_ *Q*_*n*_^+^ and *β*_*n*−1_ = (1/*n*)*y* ^*n*^ *x*_*n*_^*T*^ *Q*_*n*_^+^.

**Figure 7:**
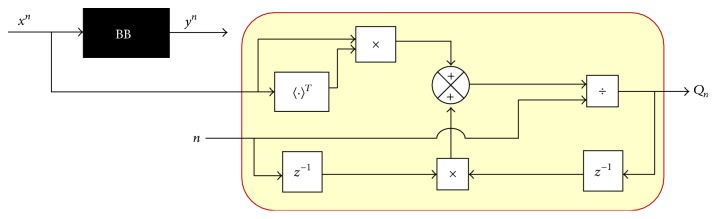
Obtainment of *Q*_*n*_, recursive.

As (31) includes (23) in its description, it is necessary to build its recursive form similar to the obtainment of (28); then we have (33) and its block diagram representation shown in Figure 7. Consider

(33) $$\begin{array}{c} Q_{n}=\left(\;\frac{1}{n}\;\right)\left[\;x^{n}x_{n}^{T}+\left(\;n-1\;\right)Q_{n-1}\;\right]. \end{array}$$

Finally, replacing (32) in (17), the identified output is the following equation:

(34) $$\begin{array}{c} y^{\text{iden},n}={\hat{A}}_{n}x^{n}. \end{array}$$

**Figure 8:**
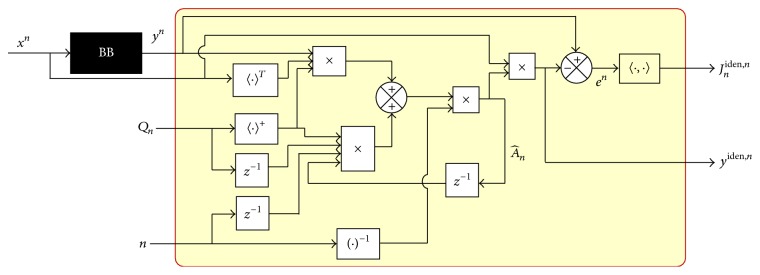
Block diagram of the analytical process.

Figure 8 represents the interaction between the inputs and the resulting error, which has better convergence due to the null error, determined for an instant by the best parameters values.

## 6. Hybrid Mechanism of Inference

**Figure 9:**
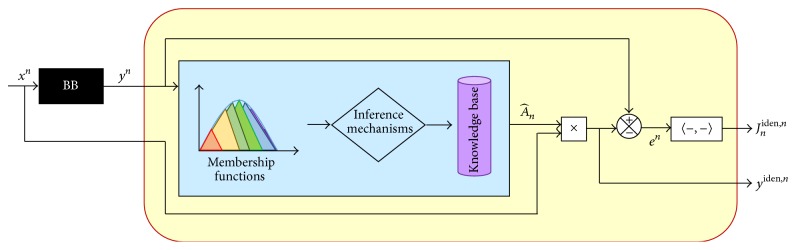
Block diagram of a hybrid filter with Fuzzy inference for an EANN model through ARMA description.

Figure 9 provides the block diagram of a hybrid filter that combines fuzzy inferences with the EANN ARMA model description, instead of the logical block from Figure 8, to determine the adequate matrix parameters. The reference model considered is a BB giving the reference signal *y* ^*n*^. The distribution curve of this signal denotes the intervals where the MF must be; then, the degree of membership obtained by Mamdani with fuzzy inferences has access to the Knowledge Base (KB), determining the parameters of the model, making the convergence, and minimizing the error in a distribution sense.

## 7. Results

The performed simulation considers a comparison between both methods giving a better idea of how they approximate to the reference signal. The reference model output *y* ^*n*^ considered nonstationary conditions, noise sequences bounded by a distribution function, and, on average, constant mean expected value and variance. The variations in the signal have a periodic signal with smooth random perturbations.

**Figure 10:**
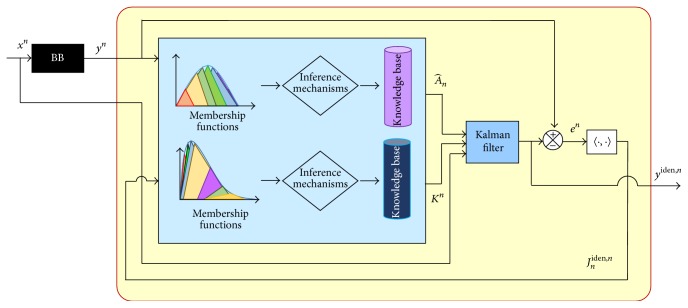
Inference process in the hybrid filter.

The first part of the simulation considered the hybrid filter, applying inferences obtaining (13) as the signal output identification.  Figure 10 shows the fuzzy inference process, where it is possible to identify the functional error given by (14), useful to estimate the coefficients for the ANN. The distribution curves defined the MFs having different operational levels represented through three and seven MFs, corresponding to *y* ^*n*^ and *J*_*n*_^*n*^, respectively.

**Figure 11:**
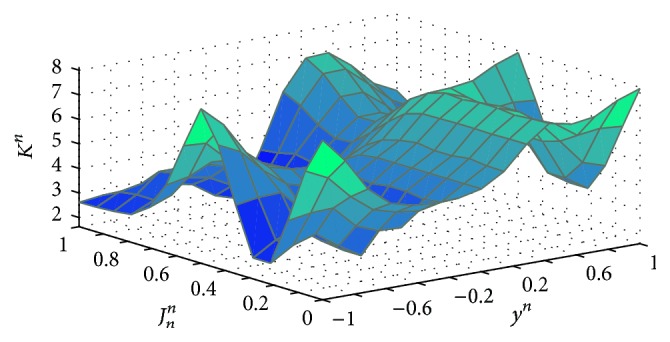
Example of a three-dimensional Knowledge Base to obtain the gain (*K* ^*n*^) through the reference signal (*y* ^*n*^) and the functional error (*J*_*n*_^*n*^).

These MFs are results of associated proper inference mechanisms to select parameters ${\hat{A}}_{n}$ and gain *K* ^*n*^ through the MFs and the KBs, affecting the final identified output ${\hat{y}}^{n}$. As an example, Figure 11 presents a three-dimensional KB integrated by sets: gain {*K* ^*n*^}, reference signal {*y* ^*n*^}, and functional error {*J*_*n*_^*n*^}. This KB helps us determine gain *K* ^*n*^ through the reference and operational error considering our expertise. The KB for ${\hat{A}}_{n}$ has a similar structure.

The analytical method uses the block diagram presented in Figure 8 having a delay in execution time due to the time state operations but within fewer process stages due to the fact that it does not require feedback from the functional error.

**Figure 12:**
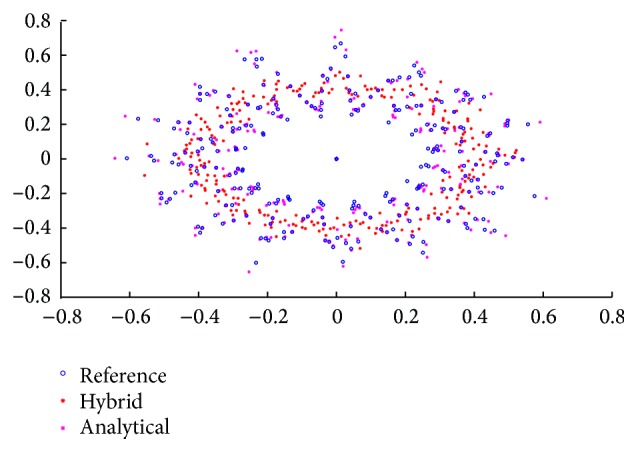
Comparison of the internal system parameters in a polar graph: reference signal parameters *A*_*n*_ (blue), estimated signal parameters (${\hat{A}}_{n}$) through hybrid estimation (red), and analytical estimation (magenta).

Our objective was to determine the internal parameters; Figure 12 compares the reference signal parameters to those estimated with both methods. The polar representation allows observing the components of the parameters; where it is possible to see, none of them leaves the unit circle.

**Figure 13 fig13:**
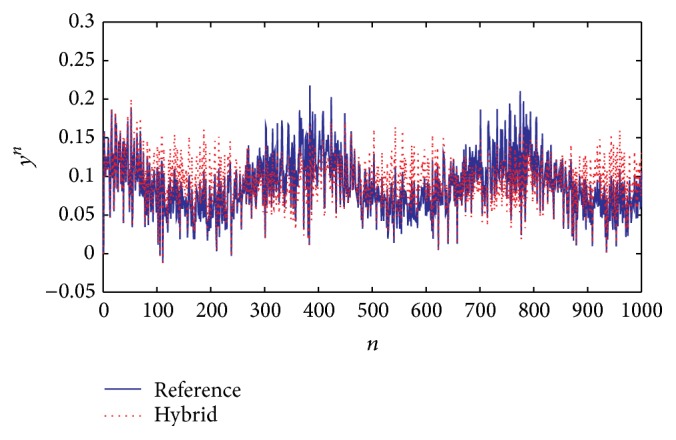
Comparison of the system response: reference signal (blue) and its hybrid approximation (red).

When applying the estimation into the hybrid system, the response is as shown in Figure 13, which presents the response following the tendency of the reference.

**Figure 14:**
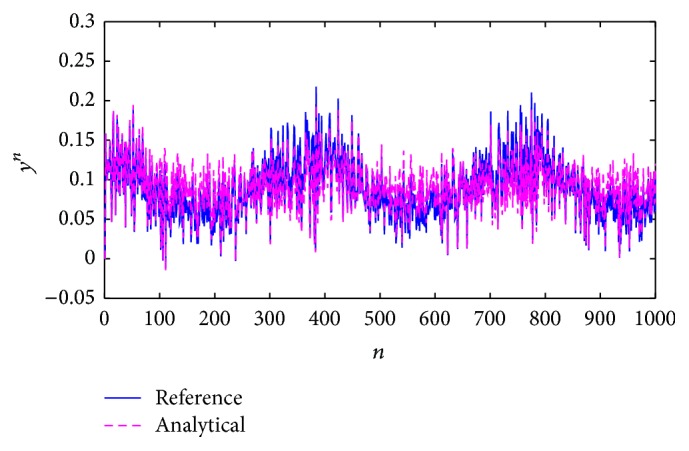
Comparison of the system response: reference signal (blue) and its analytical approximation (magenta).

The analytical method provides the response in Figure 14.

**Figure 15:**
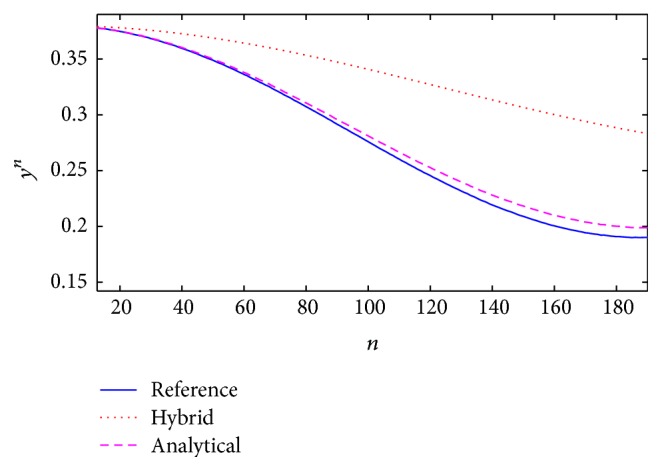
Comparison of the system response convergence for a system with variable parameters and no random noise.

The previous graphics, Figures 12 [Fig fig13]–14, were obtained considering a reference system with variable parameters and random noise. In order to better identify how the approximations converge to the reference, we have Figure 15 which presents a graphic segment showing more clearly both approximations to a reference system response, with also variable parameters but without random noise.

**Figure 16:**
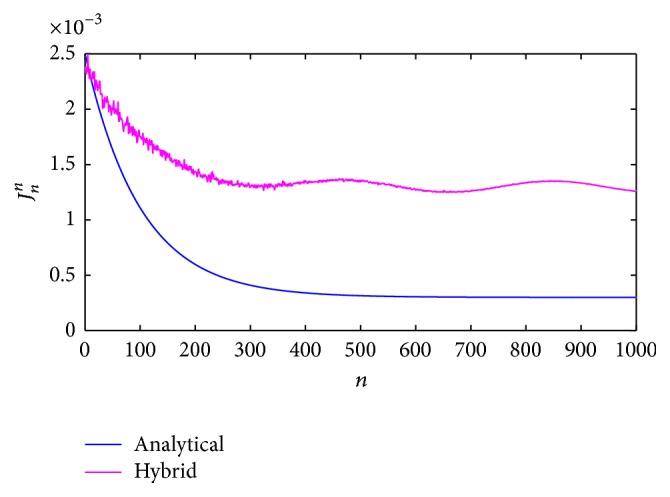
Convergence of the functional error. Comparison between the hybrid (magenta) and analytical (blue) evaluations.

From Figure 15, Figure 16 compares the convergence considering the functional error (14) from both methods. In this case, the reference is near to zero as a constant value due to the estimations considered as optimum.

## 8. Conclusion

An Equivalent Artificial Neural Net (EANN) was considered describing its parameter through a Black Box (BB) analysis using two different approximations, hybrid and analytical techniques.

For the fuzzy estimation, the best option was to consider the error properties and, in this method, the response signal was adjusted according to the reference. The fuzzy evaluation allowed the description of the coefficients and gain which affect the Kalman filter, improving the identification process according to the Multiple Inputs and Single Output (MISO) model changes with perturbations. The parameter and gain selection, using an intelligent system with classification levels, allowed selecting into the KBs the best coefficients that positively affected the filter evolution. This method does not have an exact approximation, but it is good enough on average, as shown in Figure 12, and in distribution (Figures 13 and 15) if we consider that its response converged to a particular region different from zero.

The second method used the analytical approximation, converging at almost all points to the system parameters and the reference (Figures 12 and 14) so that the expected results were a minimum functional error through time. We considered that the null error corresponded to the low energy limit, which is not zero in the neurons to avoid the total loss of connection. This method had a better approximation to the reference but achieved the minimum error only in the numerable infinite. Even though this estimation does not consider the error feedback as the first method does, its response continues being adequate when external perturbations affect the system.

A sophisticated ANN could be represented by the integration of more than one EANN due to the fact that its description allows it to consider more than one layer because it has recursive characteristics. In addition to this, the implementation of EANNs gives the possibility of having more control on the number of necessary iterations to reach a reference; this is relevant to systems where restrictions in time delays are considered essential.

Globally, both methods presented good approximations, as shown in Figure 16, with unique characteristics identifying differences between the hybrid and analytical methods.
